# Portal Hypertension Due to Hepatoportal Sclerosis in an HIV-Positive Patient Secondary to Didanosine Use

**DOI:** 10.7759/cureus.36364

**Published:** 2023-03-19

**Authors:** Davong D Phrathep, Stefan Anthony, Kevin D Healey, Hamaad Khan, Michael Herman

**Affiliations:** 1 College of Osteopathic Medicine, Lake Erie College of Osteopathic Medicine, Bradenton, USA; 2 Urology, Lake Erie College of Osteopathic Medicine, Bradenton, USA; 3 Gastroenterology, Borland Groover, Jacksonville, USA

**Keywords:** human immuno deficiency virus, hepatoportal sclerosis, esophageal and gastric varices, portal hypertension, didanosine

## Abstract

Noncirrhotic portal hypertension (NCPH) has recently been found in human immunodeficiency virus (HIV)-infected patients taking didanosine. Here, we describe an HIV-infected patient with portal hypertension due to hepatoportal sclerosis who presented with hematemesis at the emergency department (ED). CT angiography of the abdomen and pelvis with and without contrast revealed a diminutive portal vein with corresponding massive lower esophageal varices and superior mesenteric vein to the right gonadal vein varices.

Esophagogastroduodenoscopy (EGD) revealed grade II varices were found in the lower third of the esophagus, for which the patient’s symptoms improved with emergency endoscopic band ligation, octreotide and didanosine discontinuation. Our case demonstrates a rare complication that can occur with continued didanosine use in an HIV-positive patient. We highlight the need for a standard diagnostic upper gastrointestinal endoscopy to screen for portal hypertension and high-risk esophageal varices in patients with long-term didanosine use as seen in our patient.

## Introduction

Hepatoportal sclerosis (HPS) refers to the fibrosis and phlebosclerosis of portal pathways, which can lead to sinusoidal dilation and hepatic parenchymal hypoperfusion changes [1]. HPS has been shown to cause noncirrhotic portal hypertension (NCPH). The shrinking of the lumen of portal venous structures in HPS increases intrahepatic venous resistance, often manifesting clinically as variceal bleeding and elevated liver enzymes of unknown origin. Patients experiencing NCPH often have other symptoms such ascites, splenomegaly, or uncommonly hepatic encephalopathy, all of which may also be seen in cirrhosis [2]. HPS can be caused by several factors including portal vein thrombosis, nodular regenerative hyperplasia, and schistosomiasis among others, but it is notably found in HIV-positive patients taking highly active antiretroviral (HAART) medications [3]. The Food and Drug Administration (FDA) released a safety announcement stating that they acknowledge the risk of didanosine, a HAART medication, contributing to NCPH in HIV-positive patients, but the benefits of treatment outweigh this potential side effect [4]. Some researchers have also proposed that HIV itself can predispose the patient to a hypercoagulable state and increase the incidence of thrombosis, thus prophylactic measures may be considered [5,6].

## Case presentation

**Figure 1:**
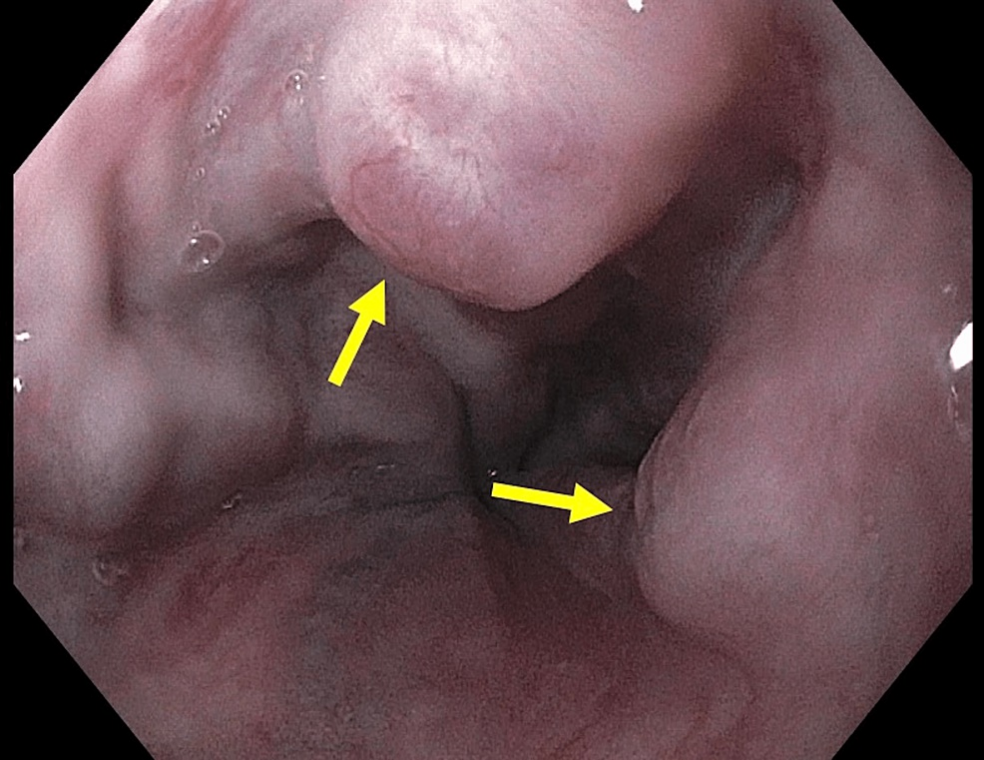
Endogastroduodenoscopy demonstrating esophageal varices in the lower third of the esophagus.

A 59-year-old female presented to the emergency department with hematemesis and dyspnea. The patient’s medical history was obtained from her family members. The patient had no prior history of gastrointestinal bleeding prior to this episode but it was reported that the patient had two episodes of melena prior to admission. The patient was not taking any non-steroidal anti-inflammatory medications at the time of admission and her family reported the patient’s past medical history positive for diabetes mellitus, gastroesophageal reflux disease, and human immunodeficiency virus. The patient’s antiretroviral medications were unknown by the family members and unable to be obtained for documentation. She did not have a history of alcohol and tobacco use. The patient's vital signs were blood pressure 118/78, temperature 98.2 degrees fahrenheit, pulse 109 beats per minute, respiration rate 22 breaths per minute, and oxygen saturation of 100% on oxygen delivery ventilator. At this time, the patient was intubated for airway protection. Physical exam revealed abdominal distension and a fluid thrill present. Due to the suspicion for esophageal varices, an endogastroduodenoscopy (EGD) was performed. On EGD, grade II varices were found in the lower third of the esophagus (Figure 1).

**Figure 2:**
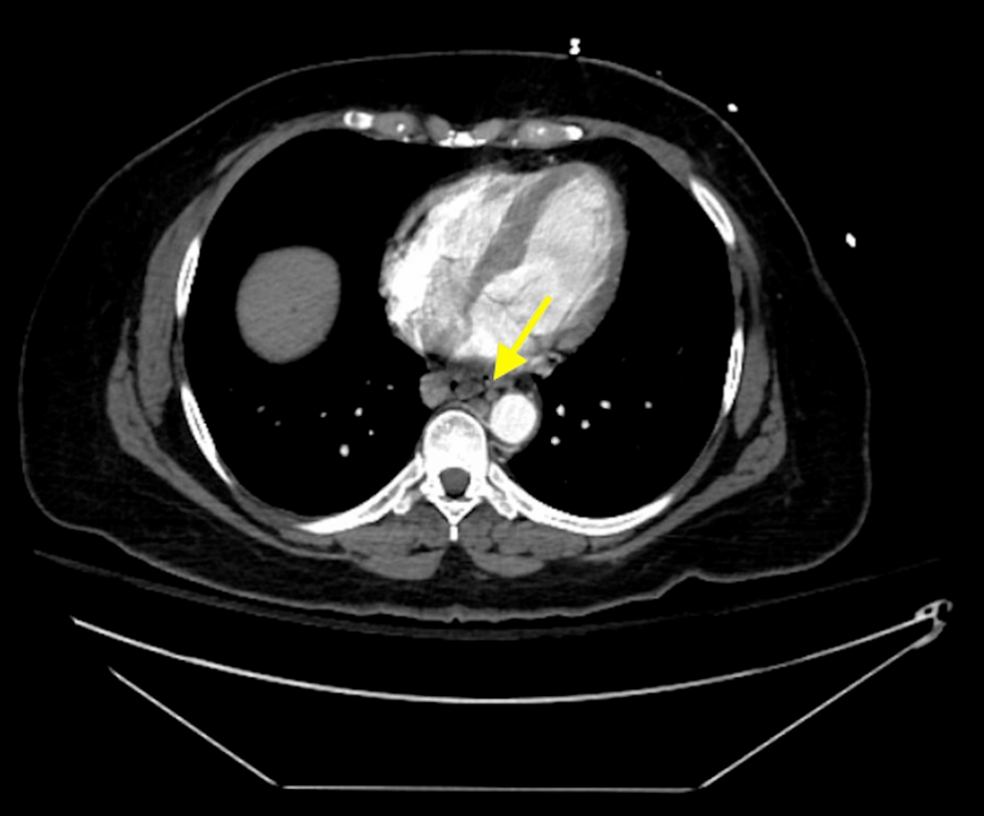
CT angiography of the abdomen and pelvis with and without contrast demonstrating lower esophageal varices.

A CT angiography of the abdomen and pelvis with and without contrast revealed a diminutive portal vein with corresponding massive lower esophageal varices (Figure 2).

Also, CT angiography of the abdomen and pelvis with and without intravenous contrast revealed superior mesenteric vein to the right gonadal vein varices. Liver biopsy revealed thickening of the walls of the portal vein and its branches and obliteration of small portal veins. There were signs of fibrosis of the tissue surrounding the portal veins. Portal hypertension secondary to cirrhosis was ruled out because liver architecture preservation. The esophageal varices were medium in size and three bands were successfully placed, resulting in deflation of varices. One of the variceal columns demonstrated a red whale sign. There was no bleeding during the procedure. Blood was found in the stomach and in the duodenal bulb. At this moment, the patient’s condition was determined to be non-cirrhotic portal hypertension due to hepatoportal sclerosis secondary to HIV. She was started on octreotide for 72 hours and ceftriaxone. It was recommended that a repeat EGD would be scheduled in two to three weeks. The patient’s condition improved and she was discharged the following day and advised to follow-up with gastroenterology for the management of her condition. On follow-up examination, the patient reported the use of didanosine for antiretroviral therapy. At this time, didanosine 400 mg once daily was discontinued and the patient was advised to begin an alternative antiretroviral therapy.

## Discussion

HPS occurs when there is thickening of the portal vein wall resulting in luminal fibrosis and obliteration [7]. The cause of HPS is due to an abnormality of the intrahepatic vasculature and is generally idiopathic [8]. However, HPS can lead to the development of noncirrhotic portal hypertension, which may clinically manifest as variceal bleed [8,9]. The majority of patients with HPS present with mild abnormalities in liver transaminases and pancytopenia due to hypersplenism [8]. HPS can result in turbulent blood flow due to the thickening of the portal vein. Loss of laminar blood flow within the hepatic system can result in an accumulation of blood within intrahepatic vessels ultimately leading to portal hypertension. Portal hypertension can lead to increased resistance to portal blood flow with increased portal vein congestion. This can cause backflow resulting in the dilation of proximal vessels leading to the development of esophageal varices as seen in our patient.

Hepatic disease is a major cause of mortality in HIV patients [10]. The cause of liver disease in this patient population is likely multifactorial. This is due to the high rates of chronic hepatitis B and C in these patients, the hepatotoxicity of antiretroviral medications and comorbidities such as alcohol, diabetes, and hyperlipidemias seen in this population [10]. HIV directly causes hepatic inflammation [10]. In current literature there are about 15 reported cases of hepatoportal sclerosis in HIV patients [11,12]. Many of these reported cases may be related to use of the antiretroviral didanosine as seen in our patient. Due to the association of didanosine with several reports of noncirrhotic portal hypertension the FDA issued an advisory in 2010 surrounding a potential hepatotoxic side effect of didanosine [11]. Uncertainty remains around how the length of exposure to didanosine correlates with its hepatotoxic qualities as the duration of didanosine therapy ranged from months to years in these reported cases [11].

Due to the high risk of upper gastrointestinal variceal bleeding, early diagnosis of non-cirrhotic portal hypertension in concordance hepatoportal sclerosis is essential. Perhaps, diagnostic upper gastrointestinal endoscopy is warranted in patients with long-term HIV and associated antiretroviral use, particularly didanosine. Additionally, the value of other less invasive screening measures for hepatoportal sclerosis such as abdominal ultrasonography or CT require further investigation. Furthermore, reduction of hepatic risk factors such as treatment of chronic hepatitis C infection should be performed when indicated to reduce the risk of portal hypertension [11]. Additionally, due to the possible complication of portal vein thrombosis, more research surrounding the efficacy of anticoagulation is warranted in these patients [11].

## Conclusions

Although portal hypertension in a patient with HIV tends to have a multifactorial etiology, we present a case report of a HIV-positive female who presented to the ED with hematemesis secondary to esophageal varices from long-standing portal hypertension and hepatoportal sclerosis. The patient's condition improved with emergent banding, octreotide and variceal treatment. We acknowledge the complex relationship between esophageal varices, portal hypertension and hepatoportal sclerosis, therefore, we reviewed didanosine’s role in causing hepatosclerosis in HIV patients. Because hepatic disease is a major cause of mortality in HIV patients, our case highlights the need for a standard prophylactic upper gastrointestinal endoscopy in patients with long-term HIV and associated didanosine use, as seen in our patient. Although it is possible that HIV contributes to the hepatic insult, we urge more research to be completed on the development of hepatic disease in an HIV-positive patient with risk factors including comorbidities, didanosine use, and infectious diseases.
